# Relationship between structural features and water chemistry in boreal headwater streams—evaluation based on results from two water management survey tools suggested for Swedish forestry

**DOI:** 10.1007/s10661-015-4385-x

**Published:** 2015-03-19

**Authors:** Ragna Lestander, Stefan Löfgren, Lennart Henrikson, Anneli M. Ågren

**Affiliations:** 1 Department of Forest Ecology and Management, Swedish University of Agricultural Sciences, 901 83 Umeå, Sweden; 2 Department of Aquatic Sciences and Assessment, Swedish University of Agricultural Sciences, P.O. Box 7050, 750 07 Uppsala, Sweden; 3 Natur och Människa AB, Friared Prästgården, 511 98 Hyssna, Sweden

**Keywords:** Water management, Forestry, Boreal forest, Eutrophication, Acidification, Siltation

## Abstract

Forestry may cause adverse impacts on water quality, and the forestry planning process is a key factor for the outcome of forest operation effects on stream water. To optimise environmental considerations and to identify actions needed to improve or maintain the stream biodiversity, two silvicultural water management tools, BIS+ (biodiversity, impact, sensitivity and added values) and Blue targeting, have been developed. In this study, we evaluate the links between survey variables, based on BIS+ and Blue targeting data, and water chemistry in 173 randomly selected headwater streams in the hemiboreal zone. While BIS+ and Blue targeting cannot replace more sophisticated monitoring methods necessary for classifying water quality in streams according to the EU Water Framework Directive (WFD, 2000/60/EC), our results lend support to the idea that the BIS+ protocol can be used to prioritise the protection of riparian forests. The relationship between BIS+ and water quality indicators (concentrations of nutrients and organic matter) together with data from fish studies suggests that this field protocol can be used to give reaches with higher biodiversity and conservation values a better protection. The tools indicate an ability to mitigate forestry impacts on water quality if the operations are adjusted to this knowledge in located areas.

**Electronic supplementary material:**

The online version of this article (doi:10.1007/s10661-015-4385-x) contains supplementary material, which is available to authorized users.

## Introduction

Freshwater resources are essential for both humans and ecosystem biodiversity and need to be sustainably used and managed in order to protect the resource. According to the EU Water Framework Directive (WFD, 2000/60/EC), all waters within the European Union should achieve a good ecological and chemical status before 2015, without further degradation of the water quality. If these requirements are not met within the timeframe, an extension to 2021 or 2027 is possible. A water body is classified into five ecological status classes (high, good, moderate, poor, or bad) based on biological, physical-chemical and hydro-morphological quality indices. Good status should resemble natural conditions, with negligible human impact on the water quality. A tangible deviation from the natural state lowers the status of a water body and requires remedial actions in order to reach good status (HVMFS 2013:19). One sector affected by the new legislation and in need for adaptation to achieve the WFD objectives is forestry (Eriksson et al. 2011).

The implementation of the WFD poses a great challenge for the forestry sector where both economy and ecological benefits should be balanced. Historically, forestry has modified running waters for timber-floating resulting in large-scale habitat degradation by decreasing habitat heterogeneity for many aquatic species (Helfield et al. 2007). Today, forestry operations such as harvesting, site-preparation, draining, ditch maintenance and construction of forest roads can result in increased runoff (Rosén 1984; Sørensen et al. 2009), erosion and sediment transport (Ahtiainen 1992; Ahtiainen and Huttunen 1999). This in turn can result in siltation of bottoms and an increased turbidity which may cause damage to aquatic species dependent on other types of substrates and clean water (e.g. Wood and Armitage 1997; Österling et al. 2010). Other forestry operations such as cutting of riparian zones (RZ) can result in biotope degradation by eliminating the ecological connection between water and land. In addition, forestry operations may lead to leakage of nutrients, base cations (Rosén et al. 1996; Piirainen et al. 2007; Löfgren et al. 2009), dissolved organic carbon (DOC) (Laudon et al. 2009; Schelker et al. 2012) and heavy metals (Bishop et al. 2009; Skyllberg et al. 2009) to surface waters. When large enough, the disturbances will have adverse effects on aquatic environments. Higher inputs of nitrogen and phosphorus can, together with reduced shading from trees, affect primary production in streams and change species composition (Holopainen and Huttunen 1992). Elevated fluxes of DOC are usually not harmful to aquatic ecosystems although it can act as a vector for mobilisation of mercury from soil to water (Kolka et al. 1999), which bioaccumulates in biota after transformation to methyl mercury (MeHg) (Garcia and Carignan 2000; Desrosiers et al. 2006). According to calculations, 9–23 % of the mercury bioaccumulation in Swedish fish can be attributed to forest harvesting operations (Bishop et al. 2009). Later research, estimating forestry effects on MeHg in Sweden, indicates that final felling annually increases the MeHg export by 14 % (Kronberg 2014). Furthermore, elevated concentrations of DOC influence pH and could potentially affect acid-sensitive aquatic organisms (Laudon and Buffam 2008). Based on base cation mass balances, there is also a fear for acidification effects coupled to intense forest production and biomass harvest (Neal et al. 1992; Akselsson et al. 2007; Ågren et al. 2010).

In Sweden, forestry has a large economic value, and 56 % (23.1 million ha) of the land cover consists of productive forests (Swedish Forest Agency 2013). More than 50,000 lakes and 290,000-km streams are located in the productive forest landscape (Ring et al. 2008), and forestry may impact the ecological and chemical status of those waters. A number of projects (Andersson et al. 2013), educational campaigns, literature reviews (Ring et al. 2008; Bishop et al. 2009), forest management optimisation models (Öhman et al. 2009; Eriksson et al. 2011) and other activities have been initiated to mitigate forestry effects on water quality. The World Wide Fund for Nature (WWF), Sweden, has, in collaboration with the Swedish forestry sector, developed two silviculture water management tools: BIS+ and Blue targeting (Bleckert et al. 2010). The tools are used to incorporate water management in the forestry planning process, so consideration is taken where it is mostly needed and where the economic outcome is least affected, i.e., balancing the economic and ecological objectives according to the Swedish forestry policy. Currently, these tools are under implementation by a number of forest companies and forest owner associations. The aims of the tools are (i) to improve the conditions for aquatic biodiversity, (ii) to optimise water consideration in Swedish forestry and (iii) to clarify the forestry sector’s responsibility for water issues.

From a WFD monitoring and classification perspective, which was never the aim for BIS+ and Blue targeting, the question arises whether this type of fairly simple and cheap field surveys of primarily structural features of a limited part of a catchment can give information on the stream water chemistry? In this study, we evaluate these two tools with the objective to evaluate the relationships between survey variables, based on BIS+ and Blue targeting data, and water chemistry in randomly selected headwater streams (*n* = 173) in the hemiboreal zone (Wallin et al. 2014; Löfgren et al. 2014). The assessment is focused on water chemical variables related to acidification and eutrophication, which are environmental quality indices in the Swedish WFD classification system (HVMFS 2013:19) and siltation. While the study has been conducted in Sweden and written from a Swedish perspective, the conclusions related to the applicability of simple silviculture water management tools are probably generalisable for much of the northern boreal region. The results from this case study are of relevance for those searching for simple water management tools related to forestry.

## Materials and methods

### BIS+ and Blue targeting

There are several methods for evaluation and classification of streams (e.g. Raven et al. 1998; SEPA 2001, 2003). The survey variables in BIS+ and Blue targets were developed as very simple tools that can be used operationally within forestry (Bleckert et al. 2010, 2011). In brief, BIS+ consist of a simple checklist (Supporting information 1) assessing a stream section’s biodiversity (B), human impact (I), sensitivity for forestry (S) and added values (+). Existence and nonexistence of mostly visible characteristics in the stream section or its RZ are marked in the checklist in the field and added to a maximum score of 12 points for each of the assessed categories (B, I, S and +). The category evaluating biodiversity (B) consists of 12 characteristics indicating a functional ecosystem with a natural biodiversity (e.g. variety in substrates and morphology, dead wood in the stream, specific habitats, threatened species and riparian shading). The absence of human impact (I) is evaluated through lack of 12 characters like migration barriers, channelisation and siltation in the stream and through assessment of land use influence on water quality and the RZ. Soil conditions and topography are determined by four indicators assessing the potential risk for erosion and rutting formation (i.e. risk of siltation) and the section’s sensitivity for forestry (S). Added values (+) comprise of other values of interest such as restorations made in the stream, other species of interest and recreational or cultural values. Many of the characteristics in BIS+ tool focus on the physical environment (morphology, dead wood, shading, etc.), but for a stream reach to be a good habitat, it also needs good water quality. Concerning water quality, there are just four indicators in the impact category that can somehow be linked to water quality: (i) siltation of the stream bottom, (ii) clear water, i.e. normal turbidity and/or colour (absorbance), (iii) acidification, which should be known beforehand, and (iv) eutrophication, i.e. local effects indicated by large amount of algae or higher vegetation.

Based on the BIS+ checklist result, the Blue target is classified for the stream section. The four different targets are VG (stream requiring general consideration), VF (stream requiring strengthened consideration), VS (stream requiring specific actions) and VO (streams to be left untouched). Blue targeting helps forest owners to optimise environmental considerations to a stream section and to identify actions needed to maintain or improve the stream biodiversity. While there is a general trend between high scores in the BIS+ protocol and a higher level of protection, there are no absolute correlation between the outcome of BIS+ and the Blue target, and instruction state that Blue target class should be judged from case to case.

### Landscape, climate and chemical variables

**Fig. 1:**
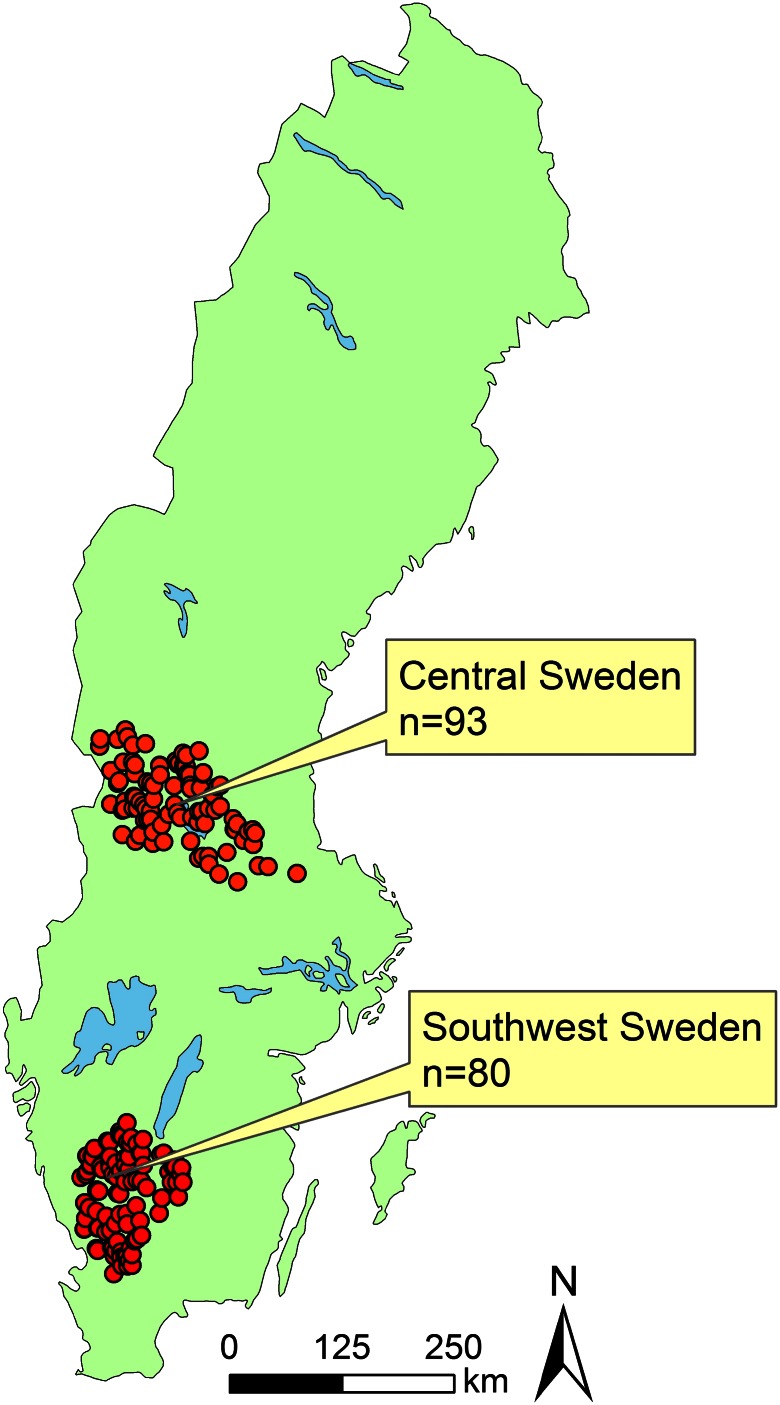
Location of sampled headwater streams in the central and southwest region of Sweden. © Lantmäteriet, i2012/901

A dataset of 173 perennial headwater streams were used for this study, whereof 80 streams were located in southwest and 93 in central Sweden (Fig. 1). The dataset contains stream water chemistry as well as landscape and climate variables at catchment level (Wallin et al. 2014; Löfgren et al. 2014). In short, headwater streams, where forest management and atmospheric deposition are the only human impact, were randomly selected from a virtual stream network constructed from a digital elevation model of 50 meter × 50 meter (Nisell et al. 2007). Stream selections were based on the criteria of a stream length > 2500 m, a distance of <500 m to a drivable road, no urban areas and <5 % agricultural land within the catchment (Göthe et al. 2013; Löfgren et al. 2014). Streams affected by liming were not included in the dataset (Löfgren et al. 2014). Landscape variables and climatic data within each catchment were calculated with information from remote sensing, satellite images, surveys from Swedish NFI (National Forest Inventory) and data from the Swedish Forest Agency, METRIA and the Swedish Meteorological and Hydrological institute (SMHI) (Göthe et al. 2013; Löfgren et al. 2014).

The pH sensitivity refers to the stream water’s ability to buffer against pH changes according to Ågren and Löfgren (2012). Stream water samples were collected at four occasions during four different seasons: spring, summer, autumn and late autumn in 2009–2011 by staff at the Swedish University of Agricultural Sciences (SLU) and the County Administrative Boards of Dalarna, Jönköping, Halland and Västra Götaland. Within 1 day of sampling, the chemical analyses following Swedish standard methods were initiated at the SWEDAC-accredited laboratory at the Department of Aquatic Sciences and Assessment, SLU (Löfgren et al. 2011, 2014). Suspended matter (SPM) was measured gravimetrically after suction filtering through a 1.2-μm glass fibre filter. Turbidity was analysed with a Turbidimeter Hach 2100AN IS at 870 nm, angle of measurement 90°. After persulphate digestion, P-tot was estimated using the molybdenium method through photometrically analysis (Bran Luebbe Autoanalyser 3). N-tot was measured using a TNMI-module equipped Shimatzu TOC-VCPH analyser. In headwater streams, TOC concentrations mostly compromise of DOC (Laudon et al. 2011). TOC concentrations in this study are therefore considered comparable to DOC concentrations in other studies.

### Study areas

The catchment characteristics for each region are presented in Table 1. The soils mostly consisted of till deposits, peat and rock outcrops, although the share of till coverage was somewhat higher in the central region. The streams were located at higher altitude in the central region compared to the southwestern region, with an average elevation of 359 m a.s.l. compared to 178 m a.s.l. A climatic gradient from south to north separated the two regions from each other with higher precipitation, run-off, mean annual temperature, forest biomass, forest growth and a longer vegetation period in the southwestern region. Norway spruce (*Picea abies*) was the dominating tree species in southwest while Scots pine (*Pinus sylvestris*) dominated in the central region. The proportion of final felled area was slightly higher in the southwest region, due to storm felling in 2005. The stream water chemistry was different between the two regions (Wallin et al. 2014; Löfgren et al. 2014) with higher concentrations of TOC, turbidity, suspended matter, N-tot and P-tot in the southwest region. Average stream pH was lower in the southwest region (pH = 5.0) compared to the central region (pH = 5.9).

**Table 1:** Catchment characteristics for the randomly selected streams in the southwestern and central regions of Sweden

	Southwest						Central					
	Mean	Min	25 %	Median	75 %	Max	Mean	Min	25 %	Median	75 %	Max
Catchment area (ha)	116	24	78	102	149	279	223	106	164	209	252	620
Forest (%)	91	37	89	96	99	100	86	54	77	88	96	100
Wetland (%)	7	0	0	2	9	59	13	0	3	11	18	46
Till (%)	57	0	42	63	76	99	70	26	59	72	82	99
Peat (%)	26	0	14	21	35	87	16	0	7	14	23	48
Rock outcrops (%)	14	0	0	2	18	83	12	0	1	6	16	66
Final felled area (%)	13.2	0.0	6.7	11.1	18.1	55.8	9.9	0.0	2.5	8.3	13.8	45.7
Elevation (m a.s.l.)	178	54	134	166	214	326	359	74	248	357	472	667
Precipitation (mm year^−1^)	995	750	950	950	1050	1250	782	650	750	750	850	950
Runoff (mm year^−1^)	501	350	450	450	550	650	424	250	350	450	450	550
Vegetation period (days)	196	190	190	200	200	210	164	140	160	160	170	180
Mean Annual Temp. (°C)	5.9	4.5	5.5	5.5	6.5	6.5	2.6	1.5	1.5	2.5	3.5	4.5
Forest biomass (kton ha^−1^)	96	22	84	97	109	146	68	30	52	65	83	110
Forest growth (m^3^ ha^−1^ year^−1^)	4.2	0.8	3.6	4.3	5.0	7.0	2.3	0.8	1.8	2.3	2.8	4.1
≥70 % Spruce (%)	23	1	16	23	29	63	11	1	6	10	15	30
≥70 % Pine (%)	11	2	7	11	15	27	25	6	17	23	34	66
NH_x_ deposition (kg ha^−1^ year^−1^)	5.4	3.5	4.5	5.4	6.2	8.0	1.5	1.1	1.3	1.5	1.6	2.1
NO_x_ deposition (kg ha^−1^ year^−1^)	5.3	4.3	5.1	5.4	5.5	5.8	2.4	1.8	2.3	2.4	2.5	3.0
SO_x_ deposition (kg ha^−1^ year^−1^)	4.8	3.8	4.6	4.9	5.1	5.7	2.0	1.3	1.8	1.9	2.0	2.8
Turbidity (FNU)	2.4	0.4	1.0	1.5	2.5	33.0	1.0	0.0	0.4	0.7	1.2	9.4
SPM (mg L^−1^)	3.4	0.0	1.0	2.0	4.2	27.4	2.2	0.0	0.6	1.3	2.5	30.7
TOC (mg L^−1^)	29.9	6.3	19.9	27.7	38.0	77.0	18.0	1.8	9.9	16.7	24.0	58.3
pH	5.0	4.0	4.5	4.8	5.3	7.5	5.9	4.2	5.4	6.0	6.5	7.2
N-tot (μg L^−1^)	845	166	585	785	1014	2833	397	62	238	353	499	2050
P-tot (μg L^−1^)	19	5	12	16	23	73	11	1	5	9	14	82

### BIS+ and Blue targeting survey

Based on the average stream length passing through an average sized final felled area of 4.4 ha (Swedish Forest Agency 2013), a stream section of 150 m upstream from the water sampling coordinates was surveyed during the summer 2013 using the BIS+ checklist and classification of Blue targets. The surveyed area corresponded to 0.1–4.5 % of the total catchment area (24–620 ha; Table 1). Characteristics difficult to determine in the field (acidification, eutrophication, endangered species, fish species, freshwater mussels and other species of interest) were collected from various databases (SLU ArtDatabanken 2011, 2014; Water Authorities 2014) and by personal contacts with the County Administrative Boards of Dalarna, Jönköping, Halland, Västra Götaland and Kronoberg.

### Water quality indicators

Water quality indicators were selected to evaluate the tools ability to assess siltation, eutrophication and acidification. Indicators of siltation constituted of average stream water concentrations of turbidity and SPM. Eutrophication indicators involved average concentrations of N-tot and P-tot and also status classification of eutrophication according to the WFD, while acidification indicators included minimum measured pH (most critical for acid-sensitive biota), average concentrations of TOC, pH sensitivity and status classification of acidification according to the WFD. In this study, pH sensitivity refers to stream water’s ability to buffer against pH changes at inputs of acid or base and is based on ANC. In high pH waters, hydrogen ions (H^+^) are neutralised by the bicarbonate system and in low pH waters, DOC or aluminium can neutralise H^+^. Poorly buffered waters with intermediate pH values (within the pH range 5–6.2) are thus most sensitive for acidification. However, depending on pH and DOC concentration, DOC can have a double-edged effect and act either as a base or as an acid (Ågren and Löfgren 2012). Status classifications of eutrophication and acidification are physical-chemical quality indices set to determine ecological status in running waters according to the WFD (HVMFS 2013:19). Eutrophication is here determined by the deviation between current P-tot concentration and a site-specific reference value of P-tot calculated as a function of concentrations of nonmarine base cations, absorbance at 420 nm and elevation above sea level for the sampling site. The status classifications of acidification were determined by the deviation between current pH (here minimum measured pH) and a site-specific reference value modelled using Model of Acidification of Groundwater In Catchments (MAGIC, Cosby et al. 2001). The model describes the dynamic development of acid–base relationships in catchments since pre-industrial time and is available from the MAGIC library database (IVL Swedish Environmental Research Institute Ltd 2013).

### Statistical analyses

The co-variance between investigated variables was tested using the multivariate method projections to latent structures by means of partial least squares (PLS) and the statistical package SIMCA-P+ version 12.0.1 (Umetrics AB, Umeå, Sweden). The intention of PLS is to reduce the dimensions in a dataset and at the same time retain as much information as possible. Each observation is projected in a multivariate X-space and a multivariate Y-space to find latent components describing the predictive variation in the variable X matrix that is linearly correlated to the positions of the response variable (Y). Models are described by the number of observations (N), cumulative fraction of X described by components (*R* ^*2*^ *X(cum)*), cumulative fraction of Y described by components (*R* ^*2*^ *Y(cum)*) and cumulative ability to predict Y with the model (*Q* ^*2*^ *(cum)*). Most important variables for explaining Y in a model are summarised in a VIP-plot (variable importance in the projection) and comprise VIP values >1. The influences of X variables on Y can be interpreted from regression coefficients (CoeffCS), which gives information of whether the influence is either positive or negative (Eriksson et al. 2006). In our PLS models, each and every water quality indicators were set as Y variables in separate models with BIS+ assessments and Blue targets set as X variables. Categorical data (Characters in BIS+, Blue targets and status classifications) were ranked and coded to numerical values to run the PLS analysis. Existence or nonexistence of an assessed characteristic in BIS+ was given a value of 1 or 0. Blue targets ranged from 1 to 4 and were ranked in the order: VG, VF, VS and VO. Ecological status classifications ranged from 1 to 5 with the value of 1 representing bad status and 5 representing high status.

**Fig. 2:**
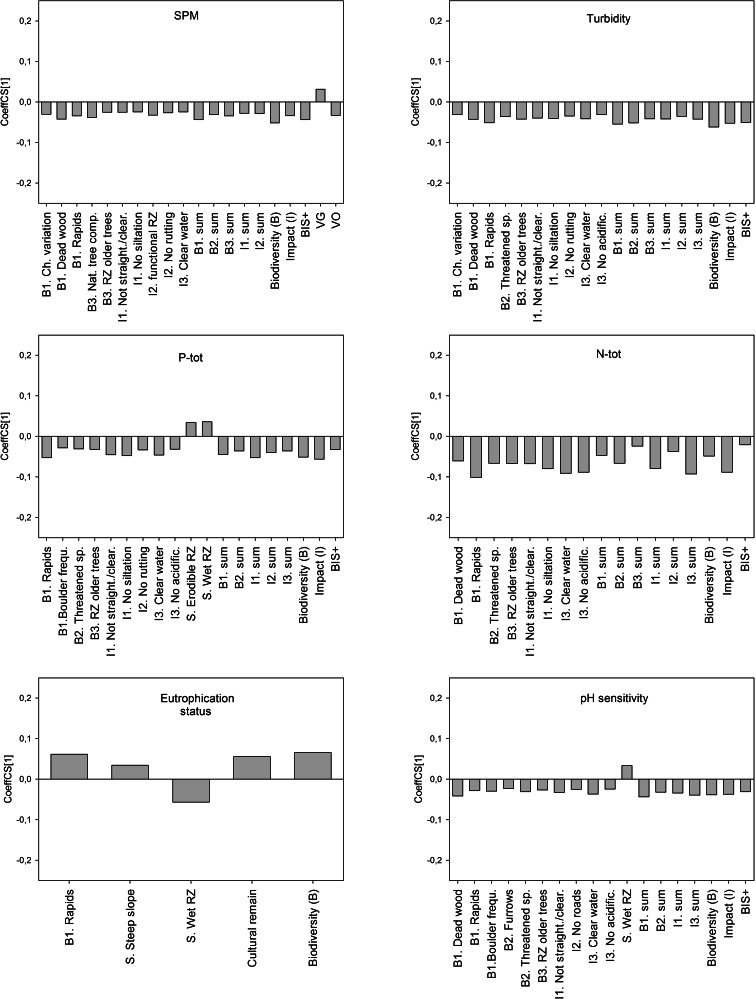

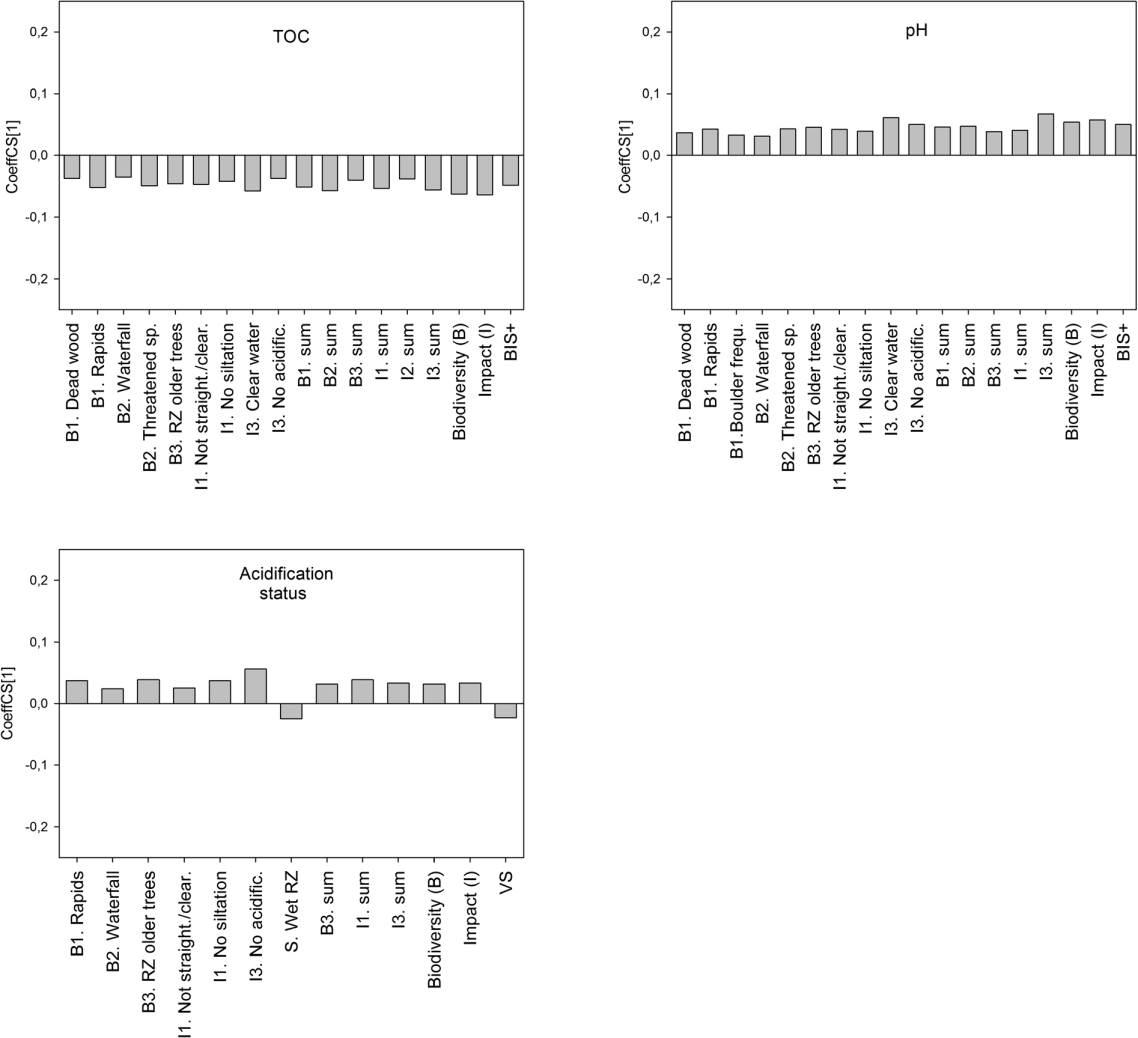
The influences (positive or negative) of variables in BIS+ and Blue targeting (X) on water quality indicators of siltation, eutrophication and acidification (Y), described by the regression coefficient (CoeffCS[1]). Only significant (*P* < 0.05) variables of importance (VIP > 1) are considered and showed in the whole dataset (both regions). For separate regional models, see Supporting Information 2

Models were tested for a combined dataset with data from both regions (Fig. 2) and for separate regional datasets (Supporting Information 2). Both X and Y variables were centred and log-transformed if necessary to fit normality before modelling. No data were excluded, although some observations may be classified as outliers.

## Results

### BIS+ and Blue targeting ability of predicting water quality indicators

The water management tools BIS+ and Blue targeting predicted the water quality indicators better when modelling data including both regions (Table 2). The predictive power, *Q* ^*2*^ *(cum)*, decreased for all investigated response variables in separate regional models (Table 2). Therefore, when we discuss the PLS models here, we relate to the analysis of the full data set e.g. both regions combined, unless stated otherwise. The chemical quality indicators turbidity, N-tot, TOC and pH were best predicted by the two water management tools in PLS models (*Q* ^*2*^ *(cum)* = 0.25, *Q* ^*2*^ *(cum)* = 0.43, *Q* ^*2*^ *(cum)* = 0.30 and *Q* ^*2*^ *(cum)* = 0.25, respectively; Table 2). However, the fraction of variation in BIS+ and Blue targeting variables (X) used to explain the best predicted response variables were low (*R* ^*2*^ *(cum) =* 0.17–0.23).

**Table 2:** PLS models of both regions and each region separately, predicting water chemical response variables regarding siltation, eutrophication and acidification (Y) using BIS+ and Blue targeting (X)

Region	Response variable (Y)	Number	*R* ^*2*^ *X(cum)*	*R* ^*2*^ *Y(cum)*	*Q* ^*2*^ *(cum)*
Siltation indicators					
Combined	SPM	173	0.17	0.18	0.09
	Turbidity	173	0.17	0.32	0.25
Southwest	SPM	80	0.11	0.21	−0.13
	Turbidity	80	0.11	0.20	−0.06
Central	SPM	93	0.13	0.25	<0.01
	Turbidity	93	0.13	0.27	0.07
Eutrophication indicators					
Combined	P-tot	173	0.16	0.24	0.16
	N-tot	173	0.23	0.54	0.43
	Eutrophication status	173	0.10	0.13	−0.12
Southwest	P-tot	80	0.13	0.22	<0.01
	N-tot	80	0.14	0.22	0.02
	Eutrophication status	80	0.12	0.27	0.01
Central	P-tot	93	0.18	0.37	−0.01
	N-tot	93	0.11	0.26	0.08
	Eutrophication status	93	0.13	0.14	−0.02
Acidification indicators					
Combined	pH sensitivity	173	0.16	0.15	0.07
	TOC	173	0.17	0.36	0.30
	pH	173	0.17	0.31	0.25
	Acidification status	173	0.15	0.09	<0.01
Southwest	pH sensitivity	80	0.10	0.18	−0.12
	TOC	80	0.14	0.27	0.09
	pH	80	0.11	0.26	−0.07
	Acidification status	80	0.11	0.16	−0.09
Central	pH sensitivity	93	0.11	0.20	−0.11
	TOC	93	0.11	0.22	<0.01
	pH	93	0.11	0.21	−0.02
	Acidification status	93	0.08	0.20	−0.06

The model displays the number of observations (N), fraction of X described by components (*R* ^*2*^ *X(cum)*), fraction of Y described by components (*R* ^*2*^ *Y(cum)*) and ability to predict Y with the model (*Q* ^*2*^ *(cum)*)

Generally, the characteristics in BIS+ (which is a more objective measure) were better predictors for stream water chemistry than the subjective Blue targets classifications (Figs. 2 and SI1). Among the water quality characteristics in BIS+ clear water, no acidification and no siltation showed significant correlations with many response variables (Fig. 2). For all eutrophication indicators, few of the characteristics in BIS+ indicating water quality were significant (Fig. 2).

#### Siltation indicators

Of the siltation indicators, turbidity was best predicted (Table 2). The PLS model for turbidity showed that the characteristics clear water, no siltation and no acidification were important variables (VIP > 1) and correlated significantly negatively (*P <* 0.05) with turbidity. Also, the characteristics clear water and no siltation in BIS+ were significant indicators for low levels of suspended material (Fig. 2). Stream water sampling showed that turbidity and SPM were higher in the southwest region and that this was captured with the BIS+ checklist.

#### Eutrophication indicators

Clear water, no acidification and no siltation were characteristics in BIS+ that significantly correlated with low concentrations of N-tot and P-tot in the PLS models (Fig. 2). This implies that stream waters in the southwestern region were assessed as more turbid (i.e. not clear), acidified and silted than in the central region, as average concentrations of stream water N-tot and P-tot were much higher in the southwest region compared to the central region (Table 1).

Another result concerning eutrophication indicators was that a wet or erodible riparian zone was of importance (*P* < 0.05) for higher stream water concentrations of P-tot, N-tot and eutrophication status according to WFD (Fig. 2, Fig. SI1).

#### Acidification indicators

Clear water, no acidification and no siltation were variables of importance for predicting lower TOC concentrations (VIP > 1, *P* < 0.05; Fig. 2) and were also significantly related to a higher pH (Fig. 2). TOC and pH were easier to predict from BIS+ (*R* ^*2*^ *Y(cum) >* 0.3) than pH sensitivity and acidification status (*R* ^*2*^ *Y(cum)* < 0.15) (Table 2). Stream sections assessed not to be acidified had higher WFD acidification status (*P* < 0.05). Lower acidification status was found to be related to wetness in the riparian zone. Stream sections surrounded by a wet riparian zone were also considered to be more pH sensitive (VIP > 1, *P* < 0.05; Fig. 2). Blue targets were of minor importance for predicting indicators of acidification.

## Discussion

### Geographical differences

The catchment characteristics vary between regions, which are reflected in the stream water chemistry (Table 1). Previous studies, analysing the stream water chemistry in the two regions, have also concluded that there are marked differences in stream water chemistry between the regions (Wallin et al. 2014; Löfgren et al. 2014). According to Löfgren et al. (2014), these differences were mostly related to climatic gradients, deposition of nitrogen and more fertile soils. Because of the larger variability in the combined dataset, the PLS models predicting water quality indicators of siltation, eutrophication and acidification had higher predictive power when modelling combined data from both regions. Subsequently, the predictability decreased in the regional models (Table 2), and the results from the separate regions should be used with caution. Greater water chemical gradients in the combined dataset were thereby easier to predict using the tools due to more pristine conditions in the central region, reflected in the field survey. Within regions, variation in stream water chemistry was probably too small to be determined with the tools. The tools have been developed with all the range of forest landscapes in mind. It would have been interesting to see if the models would have had a higher predictive power if more landscape types, with regard to climate, geology and soils, had been surveyed.

### The tools ability to predict water quality indicators

When it comes to predicting water quality indicators of siltation, eutrophication and acidification, characteristics in BIS+ were of more importance than Blue targets (Figs. 2 and SI1). The result was expected, as there is no absolute correlation between BIS+ and Blue targets, and Blue targets are subjectively chosen with support from the BIS+ checklist result, where in practicality, the sensitivity (S) to forest operation is weighed heavily in determining the Blue target class. Characteristics in BIS+ that indicates water quality will then become less important for the classification into Blue targets as all characteristics are combined. The tool BIS+ represents a more direct assessment of the stream conditions, but a more detailed analysis showed that some water quality characteristics were more important than others.

### Identification of key stream sections important for biodiversity

One of the ideas behind the BIS+ tool is that it can be used to identify stream reaches with high biodiversity, so these can be given protection. Much of the BIS+ tool focus on the physical environment (morphology, dead wood, shading, etc.) needed for the aquatic species, but for a stream reach to be a good habitat, it also needs good water quality. Of course, it is near impossible to assess water quality by just looking at a stream in the field. It is well known that stream water chemistry is a result of the characteristics in the entire catchment (Hynes 1975) and varies with climate, geology, soil type and land use (Dillon and Molot 1997; Cooke and Prepas 1998; Mattsson et al. 2003; Laudon et al. 2004; Kortelainen et al. 2006). The BIS+ only surveys the riparian zone, and while the riparian forest have been shown to have an important control on stream biogeochemistry (Lowrance et al. 2000; Grabs et al. 2012), it is also regulated on larger scales, for example by different landscape units (e.g. forest/mire) and the hydrological connectivity of those landscape units (Laudon et al. 2011). Also, only a small fraction of the entire riparian zone was surveyed in this study. Despite that, using the indicators in the BIS+ protocol, the models predicted between 20 and 30 % of the stream water quality (Table 2).

Two previous studies conducted in the southeast and northeast regions of Sweden demonstrated the BIS+ and Blue targeting tools potential for identifying stream reaches important for biodiversity (Ingemarsson 2012; Nordin 2012). The tools were evaluated by using electro fishing data, and a significantly positive relationship was noted between Blue target and fish species richness in both studies. Stream reaches assigned with targets of increased consideration levels (VO) contained more fish species than reaches assigned with targets of general consideration (VG). In addition, a positive significant correlation was found between the absence of human impact (I) and number of fish species (Ingemarsson 2012) and between the total sum of all assessed categories in BIS+ and number of species (Nordin 2012). The results of Ingemarsson (2012) and Nordin (2012) suggested that the tools can predict stream sections with higher fish species richness. These findings complement the results from our study showing correlations to water chemistry. Stream sections less affected by human impact or with a high score on the total BIS+ sum were shown to be important characteristics indicating lower concentrations of SPM, turbidity, N-tot, TOC and higher pH in most cases (Fig. 2), thereby creating a better habitat for more fish species.

### Applicability of the BIS+ protocol

In general, BIS+ models predicted between 20 and 30 % of the stream water quality, which is rather low to give specific guidance. However, there are some reflections that we want to highlight. The characteristic clear water identified stream reaches that in general were more pristine, with lower levels of TOC, turbidity, SPM, N-tot and P-tot. Negative correlations between clear water and concentrations of nutrients can be explained by the fact that N and P in boreal stream waters to a large extent are organically bound (Dillon and Molot 1997; Mattsson et al. 2003; Kortelainen et al. 2006) why less turbid and coloured waters would contain lower concentrations of nitrogen and phosphorous.

Siltation is a key variable when it comes to protecting stream biota as it can bury species underneath sediment and destroy important spawning habitats (Wood and Armitage 1997). However, we found it difficult to assess this from the field protocol instructions, where high human impact (I) is assumed to be low at normal siltation levels. But, what is normal for each site? This is impossible to know and can only be a guess. Also, the results in our study could be affected by different hydrological conditions during water chemical sampling (2009–2011) and the BIS+ and Blue targeting assessment (2013). In addition, SPM have been questioned as a reliable indicator of the siltation of bottoms (Hansen et al. 2013), as SPM tend to peak shortly after operations causing soil disturbances (Ahtiainen and Huttunen 1999; Löfgren et al. 2009) and then return back to normal levels. It was easier to assess the sensitivity (S) of the riparian soils, where the soils sensitive to erosion (and also the wet soils) correlated to high nutrient levels, indicating that it is important to protect these areas in order to mitigate nutrient leaching.

The characteristic no eutrophication in BIS+ showed little correlation with water quality indicators of eutrophication (P-tot, N-tot and WFD eutrophication status). Vegetation effects from higher input of P-tot may be difficult to detect by eye and depend on the local conditions. Whether water was anthropogenically acidified or not was determined by information from County Administrative Boards and interpolation in a water map displaying acidification of major lakes and rivers using the MAGIC model. This step needed a lot of effort and knowledge, and we feel that it is unreasonable for the average forest owner to assess whether the stream is acidified or not, and it is therefore stated in the checklist that acidification status is to be known on beforehand. Also, data of water quality status is often lacking in headwaters (Bishop et al. 2008), and the MAGIC library is not adapted to predict acidification status of headwater streams why the significantly positive correlations between the characteristic no acidification and higher WFD acidification status were somewhat unexpected. The southwestern part of Sweden is more affected by acidification from atmospheric deposition (Bertills et al. 2007), which facilitated the identification of acidified headwaters in the water map. In the central region, fewer areas were acidified on the water map making the identification of acidified headwater streams more difficult and the assessments less reliable.

To conclude, we believe that the applicability of the BIS+ tool could be improved by e.g. more detailed instructions in the form of examples supported by photos and easy access to necessary databases.

### Implications for forestry owners

For forest owners, BIS+ and Blue targeting can contribute to less forestry impact on water quality by identifying sensitive areas. Stream reaches that had a riparian zone sensitive for forestry according to BIS+ had higher concentrations of nutrients and humic substances. The results could be linked with catchment topography and the amount of wetlands in the area. The proportion of wetlands within catchments is widely recognised to be highly correlated with TOC concentration and export (Dillon and Molot 1997; Laudon et al. 2004) and can be well predicted from groundwater level and topography (Grabs et al. 2012). Kortelainen et al. (2006) showed that slope was an important variable for predicting P-tot from boreal unmanaged catchments in Finland. Both export and concentration of P-tot increased with decreasing slope (Kortelainen et al. 2006). However, the location of wetlands within the catchment could also be of importance. In the central region, stream reaches that scored high on the sensitivity (S) characteristics “wet riparian zone” and “erodible riparian zone” were often represented by riparian peat. Riparian peat is an important source of TOC in forested headwaters, where DOC concentrations generally increase upward in the soil profile (Grabs et al. 2012). This would mean that forestry operations increasing the groundwater level (e.g. harvesting) would increase TOC export (Laudon et al. 2009; Schelker et al. 2012) and organically bound nutrients to stream waters (Löfgren et al. 2009). The results suggests that stream sections identified as sensitive for forestry by BIS+ are in need of consideration in forest operation plans in order to prevent water quality deterioration.

Riparian buffer zones are a well-established best management practice in forestry (Lee et al. 2004; Thorell and Götmark 2005; Trenholm et al. 2013), where the most common practice in many parts of the world is to use a fixed width design (Lee et al. 2004). Fixed-width buffers are easily implemented and require no hydrological or ecological understanding. However, fixed-width buffers have been criticised for not accounting for the spatial heterogeneity of hydrologic pathways, biogeochemical processes and biodiversity in the riparian forests (Creed et al. 2008; Kuglerová et al. 2014). Using the BIS+ protocol, a forest owner gets a tool that allows them to plan the management of the riparian zone taking into consideration much of the knowledge from many years of underlying research of landscape structure, hydrology, biogeochemisty and ecology without needing a long formal education on these topics. Other interesting new techniques are also emerging where high-resolution maps over the riparian soils, calculated from digital elevation models (Murphy et al. 2011; Ågren et al. 2014), can be used to suggest hydrologically adapted protection zones (Kuglerová et al. 2014). Using the BIS+ tool and Blue targets is a simple way for the forest owner to prioritise the protection to where it is best needed. A better planning regarding the design of the protection zone towards water and machine-free zones may also lead to a more cost-effective hauling of timber by avoidance of rut formation in wet areas, time-consuming breakdowns, interruptions, usage of more fuel and prevention of restoration costs (Murphy et al. 2008; Mohtashami et al. 2012; Ågren et al. 2014). Thus, BIS+ and Blue targeting have the potential to improve both the biological diversity and the forest owner economic outcome.

## Conclusions

The more objective survey tool BIS+ was a better predictor for water quality than the subjective Blue target. This is hardly surprising, but it is worth noting within the community of managers and policy makers. Approximately 20–30 % of the variability in stream water quality could be assessed using the BIS+. This, in combination with studies on fish populations showing that stream reaches with high scores in the BIS+ protocol contain more fish species, lends support to the BIS+ tool as a simple way for forest owners to prioritise the location of special surface water protection in forest management. Despite the low predictive power of the models, we still argue that the tools provide a step forward compared to fixed width buffers. The characteristics in BIS+ related to the sensitivity for forestry operations in the riparian zone covary with the concentrations of nutrients and organic matter, indicating the potential of the tools to contribute to less forestry impact on the water quality if the operations are adjusted to this knowledge. The assessment methods BIS+ and Blue targeting tools may complement but cannot replace more sophisticated monitoring methods necessary for classifying water quality in streams according to WFD. This is expected, however, since their aim has never been to fulfil the WFD monitoring demands. The BIS+ protocol (Supporting Information 1) has been developed for Swedish conditions in collaboration between WWF Sweden and the Swedish forestry sector. To our knowledge, there is no similar approach directed towards the forest owner in other countries. However, simple tools like this have the potential to be implemented throughout the boreal zone after adaptation to local conditions.

## Electronic supplementary material

Below is the link to the electronic supplementary material.

ESM 1 (DOC 287 kb)

ESM 2 (DOCX 334 kb)
