# Supplementary material for: Relationship between structural features and water chemistry in boreal headwater streams—evaluation based on results from two water management survey tools suggested for Swedish forestry
Source: Environ Monit Assess. 2015 Mar 19;187(4):190. doi: 10.1007/s10661-015-4385-x (PMC4365174; doi:10.1007/s10661-015-4385-x)
Supplement: Supplementary file 1 — (DOC 287 kb) [file 10661_2015_4385_MOESM1_ESM.doc]

**Supporting Information 1.**

**Translated version of the BIS+ protocol, translated by A Ågren. Note that the translation is not an officially approved version by WWF.**

**© WWF**

**BIS+ – Assessment of Biodiversity values, Impact, Sensitivity and Added value of streams
Preliminary English version, translation by Anneli Ågren, not approved by WWF**

| **Date:** | | | | **Name of surveyor:** | | | | |
| --- | --- | --- | --- | --- | --- | --- | --- | --- |
| **Name of stream** | | | |  | | | | |
| **Catchment area** | | | | Number: | Name: | | | |
| **Stretch surveyed (m)** | | | |  | | | | |
| **Coordinates lower** | | | | X | | | Y | |
| **Coordinates upper** | | | | X | | | Y | |
| **Average width** (estimated: <1 m, <3 m, <6 m, > 6 m) | | | | | | **Dominating bottom substrate:** | | |
|  | | | | | | | | |
| ***Mark with X if present!*** | | | | | | | | |
| **B1. BIODIVERSITY VALUES – Stream** | | | | | | | | |
| Channel variation | |  | | Stream mostly meandering, large variation in depth and width, and occurrence of sand/gravel and stones/boulders. | | | | |
| Dead wood in stream | |  | | > 7 pieces per 100 m. Length of pieces > 1 m and 10 cm ∅ | | | | |
| Rapids or fast-flowing water | |  | | Distance > 10 times the average width. | | | | |
| Stretch with lots of boulders | |  | | Boulders >0,5 m ∅, distance more than 10 times the average width. | | | | |
| **Sum credits; 0 - 4** | |  | | *One X = 1 credit etc.* | | | | |
| **B2. BIODIVERSITY VALUES – Special biotopes and species** | | | | | | | | |
| Natural waterfall | |  | | Water falling in 90°, height > 1 m, often forming a natural migration barrier. | | | | |
| Braided channel | |  | | The stream splits up in > 3 channels, > 10 m length, with water all year round. | | | | |
| Inlet or outlet of lake | |  | | Not regulated. Not deepened. Position of outlet/inlet not changed by digging. | | | | |
| Valuable species | |  | | Red-listed species (should normally be know in advance of survey) or substantial occurrence or reproduction of sensitive mussels and salmonids. | | | | |
| **Sum credits; 0 - 4** | |  | | *One X = 1 credit etc.* | | | | |
| **B3. BIODIVERSITY VALUES – Riparian zone** | | | | | | | | |
| Shading exists for >75% | |  | | Riparian zone provide shading of the stream | | | | |
| Natural tree composition | |  | | Regarding the actual site, without human disturbance/forestry | | | | |
| Older trees in RZ | |  | | At the age of normal final felling, producing dead wood etc. | | | | |
| Flooded area or permanent discharge area or spring. | |  | | Periodically flooded riparian zone; to be observed from marks on the vegetation, stones, trees and ground. One large, or several obvious objects along the stretch. | | | | |
| **Sum credits; 0 - 4** | |  | | *One X = 1 credit etc.* | | | | |
| **TOTAL BIODIVERSITY VALUES** | |  | |  | | | | |
|  | | | | | | | | |
| **I1. No human IMPACT – Stream** | | | | | | | | |
| Not cleaned, and/or straightened. | |  | | Not cleaned: Stream with natural occurrence of boulders, stones and gravel.  Not straightened: Natural meandering – not straightened, not deepened | | | | |
| No siltation on bottoms | |  | | Normal amount of particles of fine material located on bottoms of gravel and sand. | | | | |
| No water regulation and/or extraction of water | |  | | No adjustment: No occurrence of one or several dams, often with an arrangement for adjustment of the water level. No removal of water: no hoses, pumps etc. in or along the stream. | | | | |
| No migration barriers | |  | | No dams, culverts, or other artificial barriers for fish or benthic fauna. | | | | |
| **Sum credits; 0 - 4** | |  | | *One X = 1 credit etc.* | | | | |
|  | | | | | | | | |
| **I2. IMPACT – Riparian zone** | | | | | | | |  |
| Functional riparian zone |  | | Ecologically functional riparian zone. No serious damage on the riparian zone, damage on < 25 % of the stretch. | | | | |  |
| No outflow from ditches |  | | No ditches flowing directly into the stream; without infiltrating through a wetland OR sediment trap. | | | | |  |
| No soil damage |  | | No old or new soil damage (ruts caused by heavy vehicles or scarification) in or along the stream which might have had a negative effect on the stream (eg. siltation). | | | | |  |
| No roads |  | | No road crosses the stream, and no road within 10 m from the stream. | | | | |  |
| **Sum credits; 0 - 4** |  | | *One X = 1 credit etc.* | | | | |  |
| **I3. IMPACT – Water quality** | | | | | | | |  |
| Clear water |  | | Normal level of turbidity and/or coloured water. | | | | |  |
| No acidification |  | | Should normally be known in advance of survey. | | | | |  |
| No eutrophication |  | | No large amounts of vegetation, for instance green algae and/or reed in the stream. | | | | |  |
| No point sources |  | | No drainage from farmland, no discharge of wastewater straight into the stream. | | | | |  |
| **Sum credits; 0 - 4** |  | | *One X = 1 credit etc.* | | | | |  |
| **TOTAL IMPACT** |  | |  | | | | |  |
|  | | | | | | | |  |
| **S. SENSITIVITY** | | | | | | | |  |
| Soil tending to erode |  | | Coarse sand, sandy or silty soils, or peat in the area. | | | | |  |
| Steep slope |  | | >5 m slope within a distance of 30 m, towards the stream | | | | |  |
| Wet-moist riparian zones |  | | Soft soils, sensitive to heavy machinery causing soil damage in or close to the stream | | | | |  |
| Spring or discharge area |  | | Water overflowing the ground and/or shallow ground water in the area | | | | |  |
| **TOTAL SENSITIVITY** |  | | *One X = 3 credit, Two X = 6 credits, etc.* | | | | |  |
|  | | | | | | | |  |
| **+. ADDED VALUE** | | | | | | | |  |
| Cultural remains |  | | Intact mills, stone foundations, timber floating arrangements, stone bridges etc. | | | | |  |
| Nature protection or Recreational area |  | | Nature reserve etc. Frequently used recreational area, for example foot paths, picnic area, signs, or area often used for fishing. | | | | |  |
| Restoration measures |  | | Liming, restoration of migration routes etc. | | | | |  |
| Interesting species |  | | For example beaver, some particular species of fish, birds and plants. | | | | |  |
| **TOTAL ADDED VALUE** |  | | *One X = 3 credit, Two X = 6 credits, etc.* | | | | |  |

| **Point object** (connected to water)**:** x: y: Type: Action: |
| --- |
|  |

**General description and comments**

*Write a general description of the stream reach and note other conditions which might effect N, P, K or* +.

|  |
| --- |
|  |
|  |

**Final assessment**

|  | **Bidiversity values** | | | **Impact** | | | **Sensitivity** | **Added**  **value** | **BIS+** | **Blue target class (VG, VF, VS, VO)** |
| --- | --- | --- | --- | --- | --- | --- | --- | --- | --- | --- |
|  | **B1** | **B2** | **B3** | **I1** | **I2** | **I3** |  | | | |
| **RESULT** |  |  |  |  |  |  |  | | | |
| **TOTAL** |  | | |  | | |  |  |  |  |
| **ASSESSMENT*** |  | | |  | | |  |  |  | |

**Actions according to Blue Target**

*Propose actions needed to improve B, I, S o*r +.

|  |
| --- |
|  |
|  |
