# Supplementary material for: Relationship between structural features and water chemistry in boreal headwater streams—evaluation based on results from two water management survey tools suggested for Swedish forestry
Source: Environ Monit Assess. 2015 Mar 19;187(4):190. doi: 10.1007/s10661-015-4385-x (PMC4365174; doi:10.1007/s10661-015-4385-x)
Supplement: Supplementary file 2 — (DOCX 334 kb) [file 10661_2015_4385_MOESM2_ESM.docx]

**Supporting Information 2.**

**Figure SI 1,** The influences (positive or negative) of variables in BIS+ and Blue targeting (**X**) on water quality indicators of siltation, eutrophication and acidification (**Y**), described by the regression coefficient (CoeffCS[1]). Only significant (*P*<0.05) variables of importance (VIP>1) are considered and showed for the whole dataset (both regions) model and for the separate regional models.
